# Rapid detection of *Pseudomonas aeruginosa* targeting the *toxA* gene in intensive care unit patients from Beijing, China

**DOI:** 10.3389/fmicb.2015.01100

**Published:** 2015-10-06

**Authors:** Derong Dong, Dayang Zou, Hui Liu, Zhan Yang, Simo Huang, Ningwei Liu, Xiaoming He, Wei Liu, Liuyu Huang

**Affiliations:** ^1^Institute of Disease Control and Prevention, Academy of Military Medical SciencesBeijing, China; ^2^Department of Digestive System, The Second Affiliated Hospital of Dalian Medical UniversityDalian, China

**Keywords:** *P. aeruginosa*, PSR, *toxA*, rapid diagnosis, isothermal

## Abstract

*Pseudomonas aeruginosa* is a major opportunistic pathogen in hospital-acquired infections and exhibits increasing antibiotic resistance. A rapid and sensitive molecular method for its detection in clinical samples is needed to guide therapeutic treatment and to control *P. aeruginosa* outbreaks. In this study, we established a polymerase spiral reaction (PSR) method for rapid detection of *P. aeruginosa* by targeting the *toxA* gene, which regulates exotoxin A synthesis. Real-time turbidity monitoring and a chromogenic visualization using hydroxynaphthol blue were used to assess the reaction. All 17 non- *P. aeruginosa* strains tested negative, indicating the high specificity of the PSR primers. The detection limit was 2.3 pg/μl within 60 min at isothermal temperature (65°C), 10-fold more sensitive than conventional PCR. Then, the PSR assay was applied to a clinical surveillance of *P. aeruginosa* in three top hospitals in Beijing, China. Of the 130 sputum samples collected from ICU patients with suspected multi-resistant infections, 37 *P. aeruginosa* isolates were identified from the positive samples. All clinical strains belonged to 10 different *P. aeruginosa* multilocus sequence typing groups and exhibited high resistance to carbapenems, cephalosporins, and aminoglycosides. Interestingly, of the 33 imipenem-resistant isolates, 30 (90.9%) had lost the outer membrane porin *oprD* gene. Moreover, isolate SY-95, containing multiple antibiotic resistance genes, possessed the ability to hydrolyze all antibiotics used in clinic and was susceptible only to polymyxin B. Our study showed the high level of antibiotic resistance and co-occurrence of resistance genes in the clinical strains, indicating a rapid and continuing evolution of *P. aeruginosa.* In conclusion, we developed a *P. aeruginosa* PSR assay, which could be a useful tool for clinical screening, especially in case of poor resources, or for point-of-care testing.

## Introduction

*Pseudomonas aeruginosa* is a common opportunistic pathogen capable of infecting both humans and animals. It causes various nosocomial diseases such as pneumonia, respiratory infection (Pereira and Cardoso, 2014), festering wounds (Altoparlak et al., 2005), urinary tract (Mittal et al., 2009), bacteremia (Al-Hasan et al., 2008), and keratitis (Hazlett, 2004). Pulmonary colonization with *P. aeruginosa* is considered a major cause of morbidity and mortality in patients with cystic fibrosis (Davies, 2002). Moreover, massive use of broad-spectrum antibiotics has increased the resistance of *P. aeruginosa* to clinical drugs, which has led to serious therapeutic problems (Peng et al., 2014). Thus, timely and accurate diagnosis is necessary for appropriate treatment and to control disease outbreaks.

Various diagnostic methods have been established for *P. aeruginosa*, including phenotypic methods (Jin et al., 2011), electrochemical techniques (Webster et al., 2014), and molecular methods such as PCR (Aghamollaei et al., 2015), real-time PCR (Qin et al., 2003; Deschaght et al., 2011), and enzyme-linked immunosorbent assay (Mauch et al., 2014). However, these methods are relatively complex, time-consuming, and require specialized, costly instruments, and expertise. Thus, a simple, cost-effective, and rapid detection method is needed.

Polymerase spiral reaction (PSR), a novel nucleic acid amplification method based on the utilization of a DNA polymerase with strand displacement activity under isothermal conditions, meets the requirements of rapidity, high sensitivity and specificity (Liu et al., 2015). Moreover, compared with other established isothermal amplification methods, PSR does not need an initial incubation at 95°C or the inclusion of a DNA helicase in the reaction mixture to achieve denaturation of the DNA double helix (Walker et al., 1992; Notomi et al., 2000; Vincent et al., 2004).

Exotoxin A is an important virulence factor of *P. aeruginosa* in clinical infections. It is a cytotoxic agent that, similar to diphtheria toxin, inhibits protein biosynthesis at the level of polypeptide chain elongation factor 2, leading to great tissue and organ damage (Jenkins et al., 2004). The *toxA* gene, an inherent genetic sequence located on the *P. aeruginosa* chromosome and regulating the synthesis of exotoxin A, has been widely used as a target for *P. aeruginosa* detection in PCR and RT-PCR methods (Khan and Cerniglia, 1994; Qin et al., 2003; Xu et al., 2004). In this study, we designed PSR primers targeting the *toxA* gene and optimized the PSR conditions. The specificity and sensitivity of PSR for detection of *P. aeruginosa* were determined. Finally, on the basis of PSR method, we molecularly characterized clinical *P. aeruginosa* isolates and investigated their dissemination in intensive care unit (ICU) patients from three top hospitals of Beijing, China.

## Materials and Methods

### Bacterial Strains, Identification, Multilocus Sequence Typing (MLST), and Antimicrobial Susceptibility Testing

A total of 56 bacterial strains were used in this study (Supplementary Material), including *P. aeruginosa* ATCC 15442 and *P. aeruginosa* CMCC 10539 as positive controls. Seventeen non- *P. aeruginosa* strains including species homologous to *P. aeruginosa* and other clinical pathogens were included to assess the specificity of the PSR assay. One-hundred and thirty clinical sputum samples were collected from ICU patients with suspected multi-resistant infections in three top hospitals of Beijing. Genomic DNA was extracted from the sputum samples using the Wizard Genomic DNA Purification Kit (Promega, Madison, WI, USA) and then subjected to the PSR assay. The bacterial strains and positive clinical samples were cultured in brain–heart infusion broth according to a standard protocol. Species identification was carried out using the Phoenix Automated Microbiology System (BD Diagnostic Systems, Franklin Lanes, NJ, USA) and sequencing of 16S ribosomal DNA (rDNA).

Multilocus sequence typing was performed to determine the sequence types (STs) of *P. aeruginosa* strains. Seven housekeeping genes, namely, *acs*A, *aro*E, *gua*A, *mut*L, *nuo*D, *pps*A, and *trp*E, were amplified using PCR. The allele sequences for each gene were compared to the *P. aeruginosa* MLST database^1^ to yield the allelic profile.

Antimicrobial susceptibility testing was performed using broth microdilution susceptibility testing according to the Clinical and Laboratory Standards Institute guidelines (CLSI, Performance Standards for Antimicrobial Susceptibility Testing; Twenty-third informational supplement CLSI Document M100-S23; Wayne, PA, USA 2013). A screening of the *oprD* gene which has a function in the *P. aeruginosa* outer membrane was performed (Wolter et al., 2004). Additionally, the strains were screened for the presence of known metallo-β-lactamase (MBL) and other β-lactamase genes (*bla*_V IM_, *bla*_IMP_, *bla*_KPC-2_, *bla*_TEM_, *bla*_SPM-1_, *bla*_SIM-1_, *bla*_NDM-1_, and *bla*_OXA-50_) using PCR with primers reported previously (Poirel et al., 2007; Farajzadeh et al., 2014).

### PSR Primer Design for the *toxA* Gene

**FIGURE 1:**
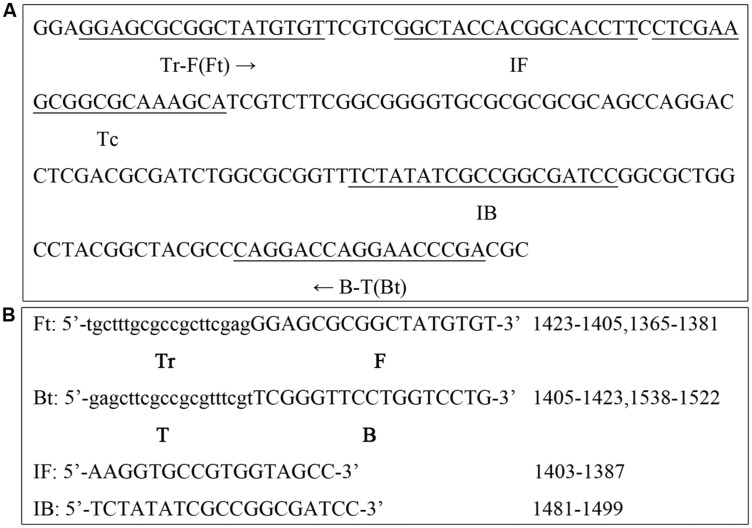
**Primer design for the polymerase spiral reaction (PSR) assay. (A)** Nucleotide sequence of the *toxA* gene (part) and locations of the primers are underlined. **(B)** Primer sequences targeting *toxA*.

To design *P. aeruginosa* specific PSR primers, the nucleotide sequence of *toxA* was downloaded from the NCBI GenBank database^2^ The primer sequences and their locations are shown in **Figure 1**. The uppercase 3′ sequences of the forward primer (F) and reverse primer (B) are complementary to the target *toxA* gene sequence (nucleotide positions 1365–1381 and 1538–1522, respectively). The lowercase 5′ sequence of the reverse primer (T) is complementary to the target sequence (position 1405–1423), and is reverse to the lowercase 5′ sequence of the forward primer (Tr). Additionally, two accelerated primers IF and IB were included in this study (positions 1403–1387, 1481–1499, respectively).

### PSR Assay

The PSR assay was carried out in 25-μl reaction mixtures containing the following components: 1.0 μl *Bst* DNA polymerase, large fragment (New England Biolabs, Ipswich, MA, USA), 2.5 μl 10× ThermoPol reaction buffer (New England Biolabs), including 20 mM Tris-HCl, 10 mM KCl, 10 mM (NH_4_)_2_SO_4_, 2 mM MgSO_4_, and 0.1% Tween 20), 0.8 M betaine (Sigma–Aldrich, Saint Louis, MO, USA), 6 mM MgSO_4_, 1.4 mM of each dNTP, and an appropriate amount of DNA template. The amount of primers needed for one reaction was 1.6 μM for Ft and Bt and 0.8 μM for IF and IB. At last, the reaction mixture was overlaid with a sealing agent (Patent: ZL201210371448.5 in China) to prevent cross contamination of samples by aerosol and the reactions were performed for 60 min at 65°C.

The PSR products were detected using two methods: turbidity monitoring with a real-time turbidimeter at 650 nm or direct visual detection with the aid of hydroxynaphthol blue (HNB), which is a metal ion indicator (Goto et al., 2009). For visual detection, 1 μl of HNB (Sigma–Aldrich) solution (0.2% mass fraction) was added to the reaction tube. A positive reaction is indicated by a color change from violet to sky blue, while a negative reaction remains violet. Each experiment was performed at least three times to ensure reproducibility.

### PCR Assay

To compare the sensitivity of PSR and the traditional PCR assay, PCRs were setup using 0.5 μM forward primer ETA-F (5′-GACAACGCCCTCAGCATCACCAGC-3′) and backward primer ETA-B (5′-CGCTGGCCCATTCGCTCCAGCGCT-3′; Khan and Cerniglia, 1994), and the same amount of DNA template in a 25-μl reaction mixture. PCR was performed using the following cycling conditions: initial PCR activation, 95°C for 10 min; amplification, 30 cycles of 95°C for 30 s, 60°C for 30 s, and 72°C for 30 s; final extension, 72°C for 7 min. The products were separated on a 1% agarose gel, stained with GelRed (Biotium, Hayward, CA, USA), and visualized under an ultraviolet transilluminator (Bio-Rad, Berkeley, CA, USA).

## Results

### Temperature Optimization of the PSR Assay

**FIGURE 2:**
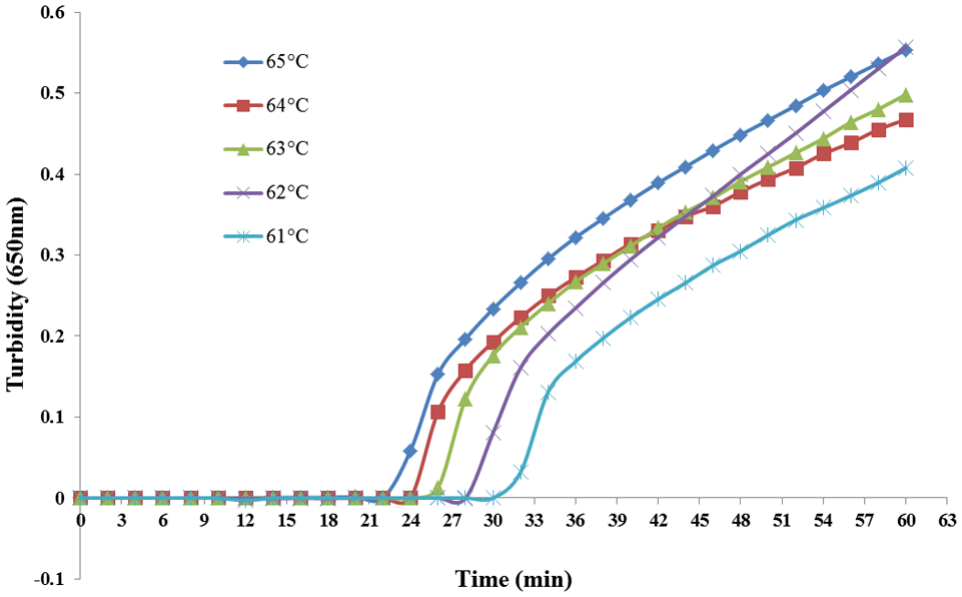
**PSR assay temperature optimization.** Different temperatures (61–65°C at 1°C intervals) were tested and 65°C was chosen as the optimal temperature for PSR amplification.

Various reaction temperatures ranging from 61°C to 65°C at 1°C intervals were compared for optimal amplification. As shown in **Figure 2**, optimal results were obtained at 65°C. It was also the optimum temperature for enzymatic activity of the *Bst* DNA polymerase.

### Specificity of the PSR Method for Detecting *P. aeruginosa*

**FIGURE 3:**
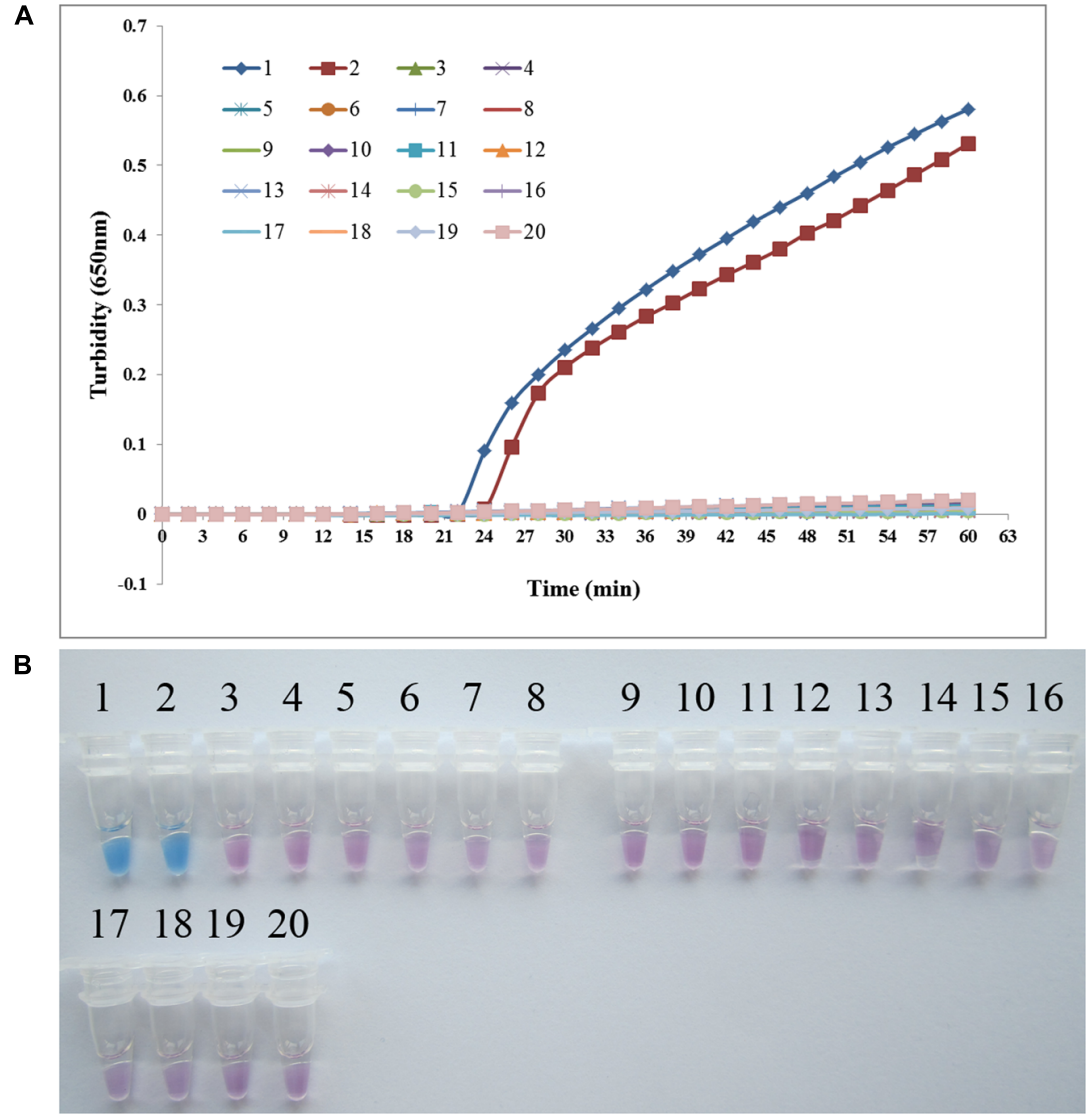
**PSR assay specificity.** Specificity of the PSR method for detecting *Pseudomonas aeruginosa* by real-time turbidimeter **(A)** or HNB colorimetric assay **(B).** Amplification was performed at 65°C for 60 min. 1, *P. aeruginosa* ATCC 15442; 2, *P. aeruginosa* CMCC 10539; 3, *P. fluorescens* CGMCC 1.1802; 4, *Burkholderia pseudomallei* 029; 5, *P. geniculate* CGMCC 1.871; 6, *P. mendocina* CGMCC 1.593; 7, *P. putida* 2309; 8, *Klebsiella pneumoniae* ATCC 2146; 9, *Streptococcus pneumoniae* 112-07; 10, *Mycobacterium tuberculosis* 005; 11, *Staphylococcus aureus* 2740; 12, *Acinetobacter baumannii* 12101; 13, *Escherichia coli* 44825; 14, *Shigella flexneri* 4536; 15, *Stenotrophomonas maltophilia* K279a; 16, *Legionella pneumophila* 9135; 17, *Haemophilus influenza* ATCC 49247; 18, *Salmonella typhi* 9275; 19, *Proteus vulgaris* CMCC 49027; 20, negative control (double distilled water).

To analyze the specificity of PSR detection of *P. aeruginosa*, genomic DNA was extracted from *P. aeruginosa* ATCC 15442, *P. aeruginosa* CMCC 10539, and 17 non- *P. aeruginosa* bacterial strains. Both real-time turbidity and visual detection of color change allowed correct identification of the *P. aeruginosa* isolates. All species homologous to *P. aeruginosa* and other clinical infectious or opportunistic strains, as well as the blank control, tested negative, indicating that the PSR assay is highly specific for *P. aeruginosa* (**Figure 3**).

### Sensitivity of the PSR Assay versus PCR for *P. aeruginosa* Detection

**FIGURE 4:**
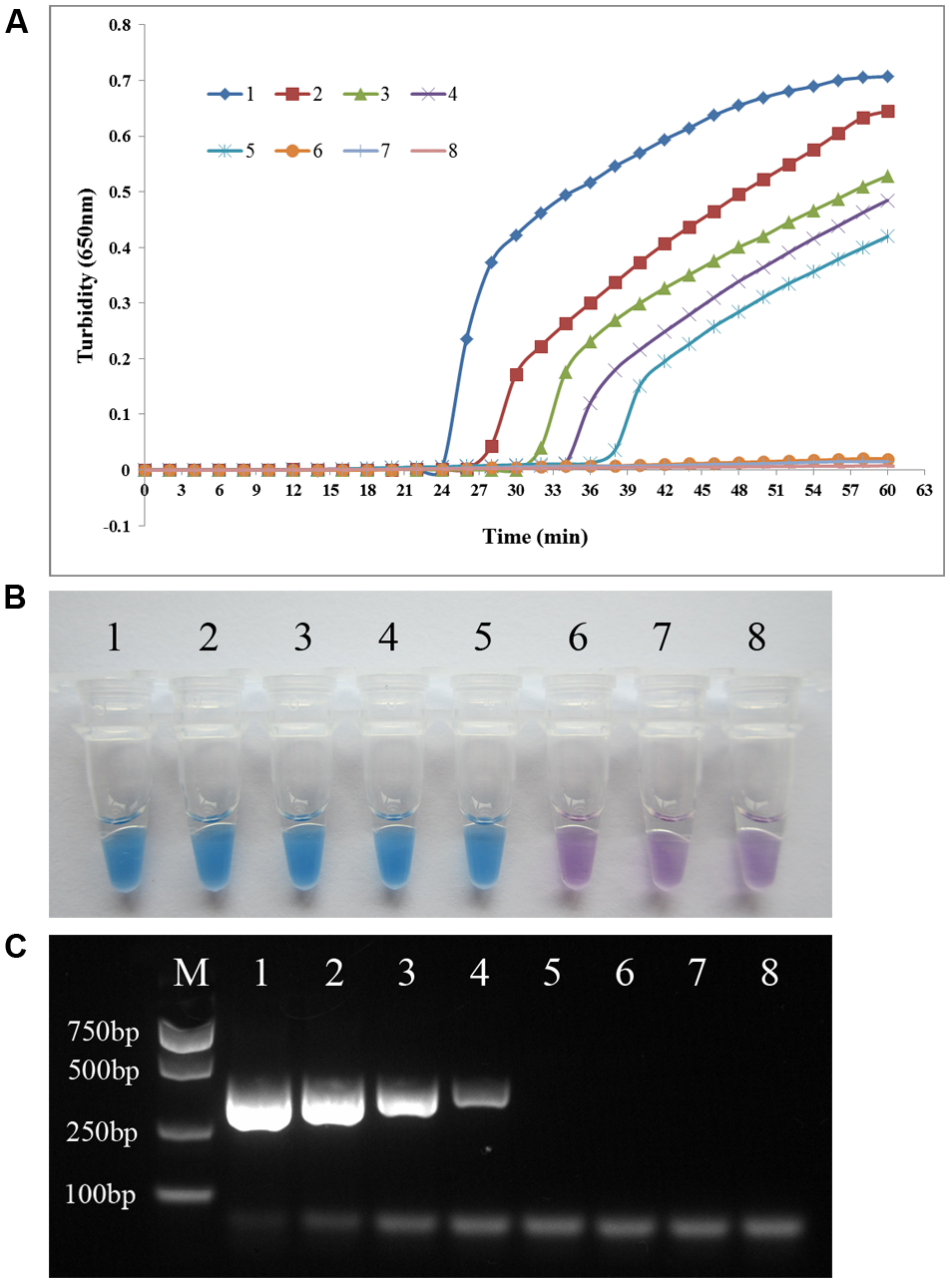
**PSR assay sensitivity.** Comparative sensitivities of the PSR assay **(A)** and **(B)** and traditional PCR **(C)** for detection of the *P. aeruginosa toxA* gene. 1–7: 10-fold serial dilution of genomic DNA extracted from *P. aeruginosa* ATCC 15442 (23.0 ng/μl to 0.023 pg/μl); 8: negative control (double-distilled water). The expected PCR product size was 396 bp.

To compare the detection limit of PSR using either real-time turbidity measurement or visual detection with that of conventional PCR, genomic DNA extracted from *P. aeruginosa* ATCC 15442 was subjected to serial 10-fold dilution (23.0 ng/μl to 0.023 pg/μl) using double-distilled water. As shown in **Figure 4**, the detection limit of both real-time turbidity and the visual method was 2.3 pg/μl, a 10-fold increase in sensitivity compared with conventional PCR.

### Dissemination of *P. aeruginosa* in Clinic

**FIGURE 5:**
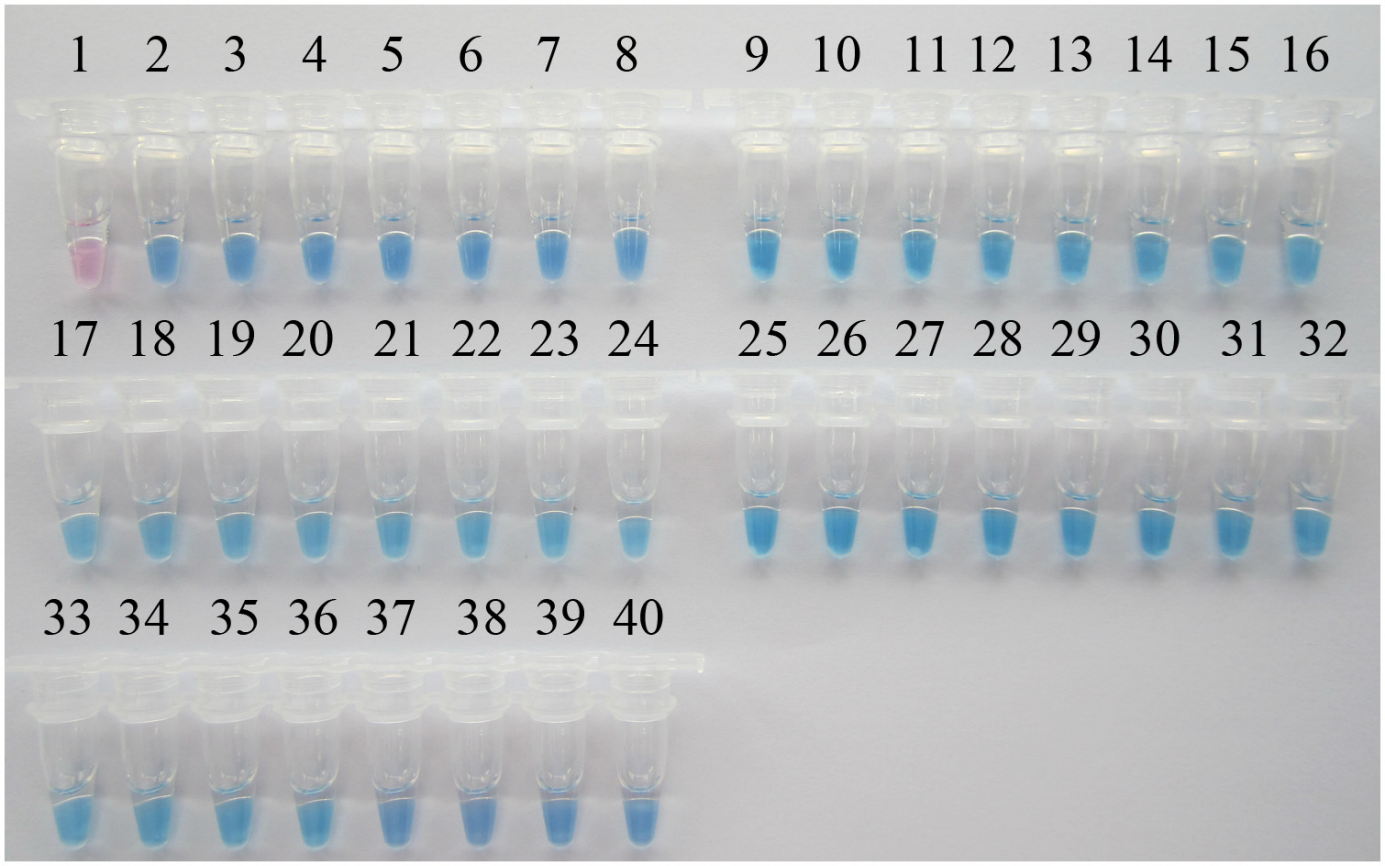
**Visual PSR detection of *P. aeruginosa* isolates from clinical samples.** The results were visualized by the addition of 1 μl HNB solution to the 25-μl reaction mix before the PSR reaction. 1, Negative control (double-distilled water); 2, positive control (*P. aeruginosa* ATCC 15442); 3, positive control (*P. aeruginosa* CMCC 10539); 4, *P. aeruginosa* SY-79; 5, *P. aeruginosa* SY-23; 6, *P. aeruginosa* SY-33; 7, *P. aeruginosa* SY-11; 8, *P. aeruginosa* SY-18; 9, *P. aeruginosa* SY-29; 10, *P. aeruginosa* SY-69; 11, *P. aeruginosa* SY-34; 12, *P. aeruginosa* SY-05; 13, *P. aeruginosa* SY-24; 14, *P. aeruginosa* SY-95; 15, *P. aeruginosa* SY-63; 16, *P. aeruginosa* SY-59; 17, *P. aeruginosa* SQ-25; 18, *P. aeruginosa* SQ-129; 19, *P. aeruginosa* SQ-09; 20, *P. aeruginosa* SQ-01; 21, *P. aeruginosa* SQ-23; 22, *P. aeruginosa* SQ-29; 23, *P. aeruginosa* SQ-37; 24, *P. aeruginosa* SQ-14; 25, *P. aeruginosa* SQ-73; 26, *P. aeruginosa* WJ-44; 27, *P. aeruginosa* WJ-66; 28, *P. aeruginosa* WJ-98; 29, *P. aeruginosa* WJ-83; 30, *P. aeruginosa* WJ-9; 31, *P. aeruginosa* WJ-26; 32, *P. aeruginosa* WJ-27; 33, *P. aeruginosa* WJ-49; 34, *P. aeruginosa* WJ-57; 35, *P. aeruginosa* WJ-41; 36, *P. aeruginosa* WJ-72; 37, *P. aeruginosa* WJ-95; 38, *P. aeruginosa* WJ-23; 39, *P. aeruginosa* WJ-01; 40, *P. aeruginosa* WJ-06.

A PSR-based surveillance of *P. aeruginosa* targeting the *toxA* gene was conducted in 301th hospital, 307th hospital, and Wujing hospital, three top hospitals of Beijing with large patient accommodations. One-hundred and thirty sputum samples were collected from ICU patients with suspected multi-resistant infections. Additionally, 10 sputum samples from healthy people were collected as controls. All clinical samples were simultaneously analyzed by visual PSR assay and traditional PCR. As shown in **Figure 5**, of the 130 clinical samples, 37 samples tested positive in the PSR assay while 34 were PCR-positive. All of the healthy control samples tested negative in each of the assays. Then, 37 *P. aeruginosa* strains were successfully cultured from the positive samples. Sequence analysis of the *toxA* gene from the isolated *P. aeruginosa* strains showed 100% identity with the nucleotide sequences reported previously (Gray et al., 1984).

Multilocus sequence typing analysis showed that the 37 *P. aeruginosa* strains belonged to different STs including ST187, ST235, ST244, ST357, ST441, ST986, ST597, ST1175, ST1224, and ST1752. Antimicrobial susceptibility testing revealed a high resistance rate of clinical *P. aeruginosa* isolates to carbapenems, cephalosporins and aminoglycosides. Only four (10.8%) strains were susceptible to imipenem. However, all clinical strains were susceptible to polymyxin B. Interestingly, mutational inactivation of *oprD* was found in 30 (90.9%) imipenem-resistant *P. aeruginosa* strains, which was consistent with a former study which claimed that reduced permeability of the outer membrane due to loss of *oprD* is the main cause of imipenem resistance (Wolter et al., 2004; Wang et al., 2010). To characterize the clinical *P. aeruginosa* isolates further, PCR screening for the presence of MBL and other β-lactamase genes (*bla*_V IM_, *bla*_IMP_, *bla*_KPC-2_, *bla*_TEM_, *bla*_SPM_, *bla*_SIM-1_, *bla*_NDM-1_, and *bla*_OXA-50_) was conducted, the results showed co-occurrence of resistance genes in most *P. aeruginosa* strains. Moreover, the isolate SY-95 containing multiple antibiotic resistance genes, presented increased resistance to all β-lactams (MIC > 128 μg/ml for meropenem and imipenem), quinolones, and aminoglycosides, and was only susceptible to polymyxin B.

## Discussion

As a non-fermentative Gram-negative bacterium, *P. aeruginosa* is currently the second most prevalent nosocomial bacterium, only after *Acinetobacter* species. *P. aeruginosa* widely exists in hospital environments such as air, water distribution systems (Loveday et al., 2014), and medical equipment (Cobben et al., 1996). According to the 2012 CHINET surveillance of antimicrobial resistance in *P. aeruginosa* in China, 71.1% of clinical *P. aeruginosa* isolates came from respiratory specimens. Infections caused by *P. aeruginosa* are notably challenging because this organism has innate resistance to a large number of antimicrobial agents. Moreover, with the acquisition of antibiotic resistance genes (such as *bla*_NDM-1_ and *bla*_IMP_), it is becoming increasingly difficult to cure infections caused by MBL-producing *P. aeruginosa* (Potron et al., 2015). Thus, early diagnosis and control of this pathogen have become increasingly important.

To meet this challenge, we established a PSR method targeting the *toxA* gene to detect *P. aeruginosa.* During the PSR reaction, a large amount of insoluble magnesium pyrophosphate as well as DNA is synthesized, resulting in increased turbidity (Liu et al., 2015). PSR amplification was monitored continuously in a real-time turbidimeter instrument or visually detected with the aid of the metal ion indicator HNB, and could be completed within 60 min with a high sensitivity of 2.3 pg/μl. The *toxA* gene regulating the synthesis of exotoxin A was targeted in the PSR assay, which did not yield false-positive amplification of species homologous to *P. aeruginosa* or of other clinical pathogens. The visual detection was as sensitive and specific as real-time turbidity measurement and further simplifies the assay, as it is visible to the naked eye. In addition, since the reaction is carried out at a constant temperature, an energy-intensive thermal cycler is not needed. A thermostatic water bath is sufficient to initiate the PSR reaction. Furthermore, the *Bst* DNA polymerase used for PSR is not influenced by the different components often present in clinical samples. Thus, DNA purification from the sample is not necessary. It is worth mentioning that the use of a wax seal to cover the reaction mixture was essential to minimize cross contamination by aerosol since the amplification efficiency of the PSR assay is extremely high, which frequently resulted in false positives.

The *P. aeruginosa* PSR assay was applied to sputum samples taken from ICU patients. The test results indicated that *P. aeruginosa* was prevalent with nearly 30% of samples tested positive. Diverse MLSTs of *P. aeruginosa* and co-occurrence of resistance genes indicated a rapid and continuing evolution of *P. aeruginosa* resulting from their wide occurrence in clinical infections, and it would be difficult to control.

Although the antibiotic resistance rate to β-lactams, aminoglycosides, and quinolones was high, all clinical *P. aeruginosa* strains were sensitive to polymyxin B. Polymyxin B is a polypeptide antibiotic of which the injectable formulation has not been introduced to China because of its potential renal toxicity. Only polymyxin B unguent for scratches and mild scalds is available. However, generalized infections need to be treated with intravenous injection or infusion. Last year, a child and his grandfather were severely burned in a fire accident in Hangzhou, China. Multiple antibiotics were invalid for the treatment, and multi-drug resistant *P. aeruginosa* strains were isolated from their wounds. It was not until the local newspaper launched a nationwide search that the required dose of polymyxin B was collected and the two patients were cured. Therefore, we think there’s a need for an increase in the reserves of this medicine in Chinese hospitals, especially those specialized in burns, as the last resort for severe injuries.

## Conclusion

We established a PSR detection method for *P. aeruginosa*, which meets the ASSURED criteria (affordable, sensitive, specific, user friendly, robust and rapid, equipment free and deliverable) proposed by the World Health Organization for developing diagnostic techniques (Mabey et al., 2004). We anticipate its routine use in clinical screening and on-site diagnosis, particularly in situations where resources are limited. Furthermore, our study provides new insights into the mechanisms of *P. aeruginosa* antibiotic resistance and warns that future therapeutic options may be seriously limited.

## Conflict of Interest Statement

The authors declare that the research was conducted in the absence of any commercial or financial relationships that could be construed as a potential conflict of interest.

## Footnotes

1 http://pubmlst.org/paeruginosa

2 ncbi.nlm.nih.gov/gene/877850
