# Supplementary material for: Rapid detection of Pseudomonas aeruginosa targeting the toxA gene in intensive care unit patients from Beijing, China
Source: Front Microbiol. 2015 Oct 6;6:1100. doi: 10.3389/fmicb.2015.01100 (PMC4594016; doi:10.3389/fmicb.2015.01100)
Supplement: Supplementary file 1 [file Table_1.DOCX]

**Supplementary Materials**

**Table 1:** Bacterial strains used in the study

| Strains | Source |
| --- | --- |
| *Pseudomonas aeruginosa* ATCC 15442 | Our microorganism center |
| *P. aeruginosa* CMCC 10539 | Our microorganism center |
| *Pseudomonas fluorescens* CGMCC 1.1802 | Our microorganism center |
| *Burkholderia pseudomallei* 029 | Our microorganism center |
| *Pseudomonas geniculate* CGMCC 1.871 | Our microorganism center |
| *Pseudomonas mendocina* CGMCC 1.593 | Our microorganism center |
| *Pseudomonas putida* 2309 | Our microorganism center |
| *Klebsiella pneumoniae* ATCC 2146 | Our microorganism center |
| *Streptococcus pneumoniae* 112-07 | Our microorganism center |
| *Mycobacterium tuberculosis* 005 | Our microorganism center |
| *Staphylococcus aureus* 2740 | Our microorganism center |
| *Acinetobacter baumannii* 12101 | Our microorganism center |
| *Escherichia coli* 44825 | Our microorganism center |
| *Shigella flexneri* 4536 | Our microorganism center |
| *Stenotrophomonas maltophilia* K279a | Our microorganism center |
| *Legionella pneumophila* 9135 | Our microorganism center |
| *Haemophilus influenza* ATCC 49247 | Our microorganism center |
| *Salmonella typhi* 9275 | Our microorganism center |
| *Proteus vulgaris* CMCC 49027 | Our microorganism center |
| *P. aeruginosa* SY-79 | Clinical isolate |
| *P. aeruginosa* SY-23 | Clinical isolate |
| *P. aeruginosa* SY-33 | Clinical isolate |
| *P. aeruginosa* SY-11 | Clinical isolate |
| *P. aeruginosa* SY-18 | Clinical isolate |
| *P. aeruginosa* SY-29 | Clinical isolate |
| *P. aeruginosa* SY-69 | Clinical isolate |
| *P. aeruginosa* SY-34 | Clinical isolate |
| *P. aeruginosa* SY-05 | Clinical isolate |
| *P. aeruginosa* SY-24 | Clinical isolate |
| *P. aeruginosa* SY-95 | Clinical isolate |
| *P. aeruginosa* SY-63 | Clinical isolate |
| *P. aeruginosa* SY-59 | Clinical isolate |
| *P. aeruginosa* SQ-25 | Clinical isolate |
| *P. aeruginosa* SQ-129 | Clinical isolate |
| *P. aeruginosa* SQ-09 | Clinical isolate |
| *P. aeruginosa* SQ-01 | Clinical isolate |
| *P. aeruginosa* SQ-23 | Clinical isolate |
| *P. aeruginosa* SQ-29 | Clinical isolate |
| *P. aeruginosa* SQ-37 | Clinical isolate |
| *P. aeruginosa* SQ-14 | Clinical isolate |
| *P. aeruginosa* SQ-73 | Clinical isolate |
| *P. aeruginosa* WJ-44 | Clinical isolate |
| *P. aeruginosa* WJ-66 | Clinical isolate |
| *P. aeruginosa* WJ-98 | Clinical isolate |
| *P. aeruginosa* WJ-83 | Clinical isolate |
| *P. aeruginosa* WJ-9 | Clinical isolate |
| *P. aeruginosa* WJ-26 | Clinical isolate |
| *P. aeruginosa* WJ-27 | Clinical isolate |
| *P. aeruginosa* WJ-49 | Clinical isolate |
| *P. aeruginosa* WJ-57 | Clinical isolate |
| *P. aeruginosa* WJ-41 | Clinical isolate |
| *P. aeruginosa* WJ-72 | Clinical isolate |
| *P. aeruginosa* WJ-95 | Clinical isolate |
| *P. aeruginosa* WJ-23 | Clinical isolate |
| *P. aeruginosa* WJ-01 | Clinical isolate |
| *P. aeruginosa* WJ-06 | Clinical isolate |
