# Supplementary material for: Rapid detection of Pseudomonas aeruginosa targeting the toxA gene in intensive care unit patients from Beijing, China
Source: Front Microbiol. 2015 Oct 6;6:1100. doi: 10.3389/fmicb.2015.01100 (PMC4594016; doi:10.3389/fmicb.2015.01100)
Supplement: Supplementary file 2 [file Table_2.DOCX]

**Supplementary Materials**

**Table 2:** Antibiotic susceptibilities minimum inhibitory concentrations (mg/L) of clinical *P. aeruginosa* isolates

| Isolate No. | IMP | MEM | BIP | CTZ | FEP | C/S | LEV | CIP | AMK | FOS | PB |
| --- | --- | --- | --- | --- | --- | --- | --- | --- | --- | --- | --- |
| SY-79 | 32 | 32 | 32 | ≥64 | 64 | 128 | 8 | 4 | 4 | 16 | 0.5 |
| SY-23 | 64 | 64 | 32 | ≥64 | 64 | 128 | 16 | 4 | 4 | 32 | 1 |
| SY-33 | 32 | 8 | 16 | 32 | 64 | 32 | 16 | 8 | ≥64 | 64 | 1 |
| SY-11 | 32 | 64 | 16 | 32 | 32 | 128 | 4 | 1 | 4 | ≥128 | 2 |
| SY-18 | 4 | ≤0.25 | ≤0.25 | 1 | 4 | 2 | 0.25 | 2 | 2 | 1 | 0.5 |
| SY-29 | 32 | 4 | 16 | 2 | 8 | 4 | 0.5 | 2 | 4 | 64 | 2 |
| SY-69 | 32 | 16 | 16 | 32 | 64 | 64 | 4 | 4 | 4 | ≥128 | 1 |
| SY-34 | 32 | 8 | 32 | 32 | 32 | 16 | 8 | 2 | ≥64 | 64 | 1 |
| SY-05 | 32 | 16 | 8 | 8 | 16 | 32 | 32 | 16 | 8 | 32 | 1 |
| SY-24 | 32 | 8 | 16 | 4 | 8 | 8 | 0.5 | 1 | 4 | 64 | 1 |
| SY-95 | ≥128 | ≥64 | ≥64 | ≥64 | ≥128 | ≥128 | ≥32 | ≥32 | ≥64 | 64 | 1 |
| SY-63 | 4 | ≤0.25 | ≤0.25 | 1 | 2 | 2 | 0.25 | 2 | 2 | 1 | 0.5 |
| SY-59 | 8 | 8 | 2 | 8 | 16 | 16 | 1 | 0.25 | 1 | 64 | 1 |
| SQ-25 | 32 | 32 | 4 | ≥64 | ≥64 | ≥128 | 1 | 4 | ≥64 | 32 | 1 |
| SQ-129 | 32 | 16 | 32 | 16 | 16 | 16 | 8 | 2 | 4 | 64 | 1 |
| SQ-09 | 32 | 32 | 16 | ≥64 | ≥64 | 128 | 8 | 2 | ≥64 | 32 | 1 |
| SQ-01 | 64 | 16 | 32 | 16 | 16 | 32 | 8 | 4 | 8 | 32 | 1 |
| SQ-23 | ≥128 | ≥64 | >64 | 64 | ≥128 | ≥128 | 0.5 | 4 | 64 | 32 | 1 |
| SQ-29 | 16 | ≥64 | 16 | 64 | ≥128 | ≥128 | 8 | 2 | 64 | 128 | 1 |
| SQ-37 | 64 | 32 | 32 | ≥64 | 64 | 8 | 1 | 2 | 32 | 64 | 1 |
| SQ-14 | 8 | 64 | 2 | ≥64 | 64 | 64 | 4 | 2 | 64 | ≥128 | 1 |
| SQ-73 | ≥128 | 32 | 64 | 64 | 64 | 128 | 8 | 4 | ≥64 | 32 | 1 |
| WJ-44 | 8 | 16 | 0.5 | 64 | 128 | 128 | 0.5 | 1 | 64 | 32 | 1 |
| WJ-66 | 128 | 32 | 16 | 64 | 128 | 128 | 0.25 | 4 | 64 | 64 | 1 |
| WJ-98 | 32 | 32 | 16 | 64 | 32 | ≥128 | 8 | 4 | ≥64 | 128 | 1 |
| WJ-83 | 32 | 16 | 4 | 16 | 32 | 128 | 8 | 4 | 16 | 32 | 1 |
| WJ-9 | 32 | 8 | 4 | 8 | 16 | 32 | 2 | 0.5 | 4 | 32 | 2 |
| WJ-26 | 64 | 8 | 32 | 32 | 16 | 16 | 8 | 0.25 | 4 | 64 | 1 |
| WJ-27 | 32 | 4 | 16 | 2 | 4 | 4 | 0.5 | 1 | 4 | 64 | 2 |
| WJ-49 | 4 | 1 | 0.5 | 2 | 8 | 16 | 0.5 | 2 | 64 | 32 | 2 |
| WJ-57 | 64 | 16 | 4 | 64 | 64 | ≥128 | 0.25 | 2 | 32 | 32 | 1 |
| WJ-41 | 64 | 16 | 4 | 64 | 64 | ≥128 | 8 | 4 | 32 | 32 | 1 |
| WJ-72 | 32 | 16 | 4 | 64 | 32 | 128 | 0.5 | 2 | 32 | 32 | 1 |
| WJ-95 | 8 | 16 | 4 | ≥64 | 64 | ≥128 | 8 | 4 | ≥64 | 64 | 1 |
| WJ-23 | 4 | 4 | 4 | 32 | 16 | 32 | 0.25 | 2 | 4 | 64 | 1 |
| WJ-01 | 64 | 16 | 32 | 16 | 16 | 32 | 8 | 4 | ≥64 | 32 | 1 |
| WJ-06 | 32 | 16 | 4 | 16 | 32 | 128 | 8 | 4 | 16 | 32 | 1 |

IMP, imipenem; MEM, meropenem; BIP, biapenem; CTZ, ceftazidime; FEP, cefepime; C/S, cefoperazone-sulbactam; LEV, levofloxacin; CIP, ciprofloxacin; AMK, amikacin; FOS, fosfomycin; PB, polymyxin B.
